# Bilateral proximal deep vein thrombosis and COVID‐19 in a patient with absence of inferior vena cava: A case report and review of literature

**DOI:** 10.1002/ccr3.5972

**Published:** 2022-06-13

**Authors:** Yaser Jenab, Parham Ghafouri, Kaveh Hosseini, Shapour Shirani, Mahmood Shirzad

**Affiliations:** ^1^ Tehran Heart Center Tehran University of Medical Sciences Tehran Iran; ^2^ School of Medicine Tehran University of Medical Sciences Tehran Iran; ^3^ Students' Scientific Research Center Tehran University of Medical Sciences Tehran Iran

**Keywords:** absence of inferior vena cava, anticoagulants, deep vein thrombosis, inferior vena cava agenesis, inferior vena cava atresia

## Abstract

Bilateral proximal deep vein thrombosis (DVT) in the lower extremities of young patients should raise suspicion over pro‐thrombotic conditions and venous anatomical abnormalities, even in the presence of other precipitating factors, such as viral infection. The authors present a 33‐year‐old man with bilateral DVT and absence of inferior vena cava (AIVC), who also had concurrent COVID‐19, and discuss the management of this patient.

## 1 INTRODUCTION

Venous thromboembolism (VTE) is the third most frequent cause of cardiovascular mortality. This condition mostly occurs in the presence of risk factors that can be acquired or congenital.^1^ Deep vein thrombosis (DVT) has an incidence as low as 1 per 10,000 in younger patient groups whereas, in the general population, the incidence rate is 1 per 1000.^2^ A recent study demonstrates that DVT has an incidence rate of 7.4% (95% CI: 3.2%–16.2%) among patients with COVID‐19, who did not require admission in the intensive care unit (ICU).^3^ Absence of inferior vena cava (AIVC) accounts for up to 5% of cases among young adults with unprovoked DVT.^4^, ^5^ Indeed, the presence of other risk factors, like COVID‐19, probably increases the incidence of DVT in AIVC.^6^ Although COVID‐19 is a major cause for VTE during the global pandemic, we must consider other risk factors. Young adult male patients with bilateral DVTs point to the diagnosis of anatomical abnormalities. Herein, we present a case with AIVC and bilateral DVT who also had concurrent severe acute respiratory syndrome coronavirus 2 (SARS‐CoV‐2) infection.

## 2 CASE PRESENTATION

A 33‐year‐old man was referred to our tertiary center, with a history of extensive bilateral DVT 6 weeks ago during admission in another center. At that time, he had mild to moderate COVID‐19 with a positive PCR test and O_2_ saturation levels ≥94% in the room air. However, because of concurrent COVID‐19 infection, he was discharged from that center on Rivaroxaban 15 mg q12 hours. Two days after, he was re‐admitted to the same hospital due to severe pain and cyanosis of both lower extremities. This time, he received low‐molecular‐weight heparin (LMWH) and warfarin for anticoagulation and prevention of thrombosis recurrence. LMWH had been prescribed within the therapeutic dose of 40 mg daily. Warfarin was also prescribed within therapeutic dose after proper bridging period with LMWH and had been continued with an INR between 2.0 and 3.0.

After admission to our center, on the initial examination, vital signs were stable and lower extremity pulses were normal. No sign of phlegmasia was noted. He had complaints of pain in both his legs but no significant edema. At this time, because of subacute course of the disease (more than 14 days), he was no longer a candidate for thrombolytic therapy.

From the risk factors of DVT, he only has a history of COVID‐19, as an inflammatory condition, but did not mention any recent immobilizations, trauma, long‐range flights, use of anabolic steroids, chest pain, or shortness of breath. There was no evident history of any thrombotic events (DVT, PTE) in other family members. He reported no use of alcohol, tobacco, and recreational drugs.

### 2.1 Investigations

On his admission, we asked for a SARS‐CoV‐2 PCR test which came back negative. Prolonged activated partial thromboplastin time (aPTT = 49.8 s) and increased international normalized ratio (INR = 2.02) can be explained in the setting of rivaroxaban intake. The liver aminotransferases (ALT = 158 IU/L [Normal range: up to 41 IU/L in males] and AST = 113 IU/L [Normal range: up to 40 IU/L in males]) were also increased. We assessed the patient for other possible causes of early DVT and thrombophilia states such as Lupus anticoagulant, anti‐Cardiolipin, anti‐β2‐glycoprotein, anti‐thrombin III, and Factor V Leiden which all became negative.

There was no imaging available from the first episode of DVT in 6 weeks ago. However, this time, two Color Doppler Ultrasound studies were performed for the patient: one in a local hospital and the other in our center. Both of them showed that external iliac, common femoral, femoral, and popliteal veins were occluded with thrombus in the right leg suggestive of acute obstructive DVT. The left common femoral and proximal part of deep femoral and femoral veins were patent and without thrombosis. However, the distal segment of the left femoral vein and popliteal vein were occluded with thrombosis (acute DVT). Thrombi also existed in the left external Iliac vein and IVC, which extends up to the liver. Intrahepatic veins and intrahepatic IVC have a normal appearance. Moreover, transthoracic echocardiography showed no evidence of thrombosis in the supra‐hepatic segment of IVC.

**FIGURE 1:**
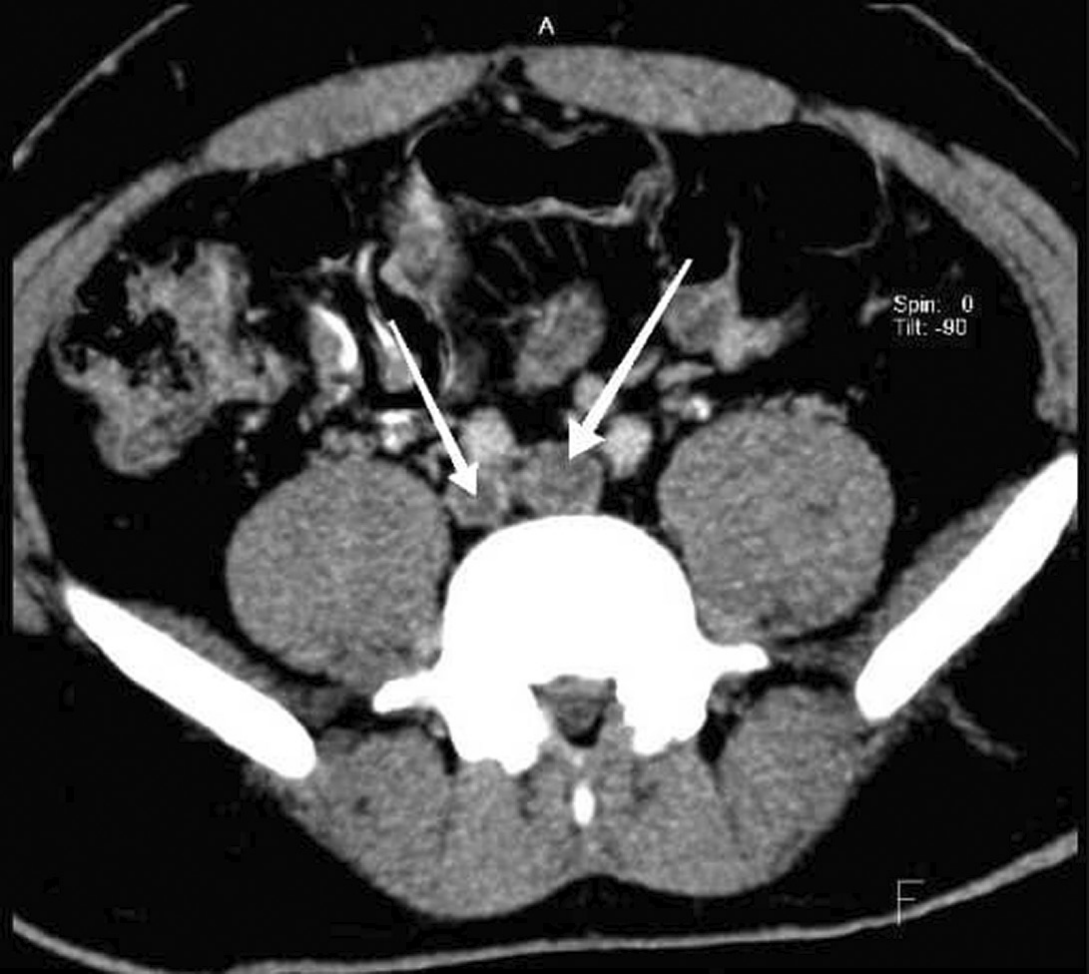
Axial CT delayed phase images, recent thrombosis in both common iliac veins (arrows)

**FIGURE 2:**
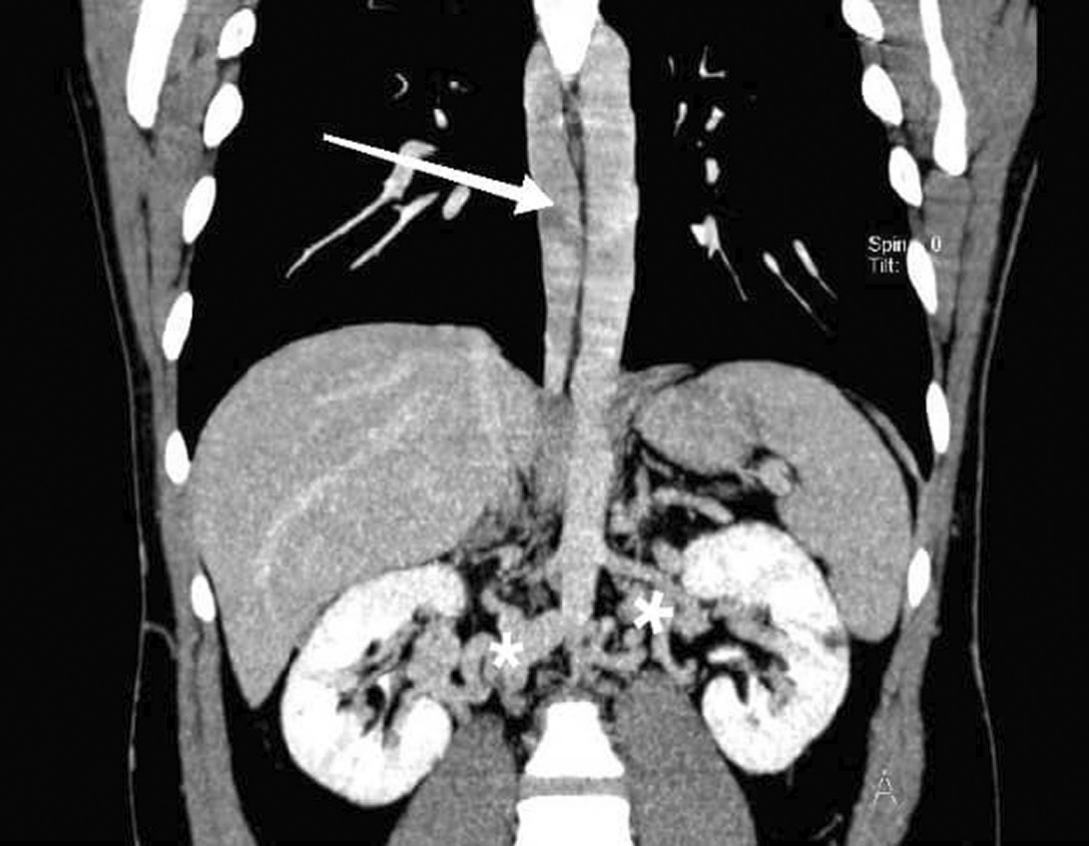
Coronal maximum intensity projection computed tomography image shows dilated azygos vein (arrow) in right side of vertebral column and multiple venous collaterals in hilum of kidneys (asterisks)

**FIGURE 3:**
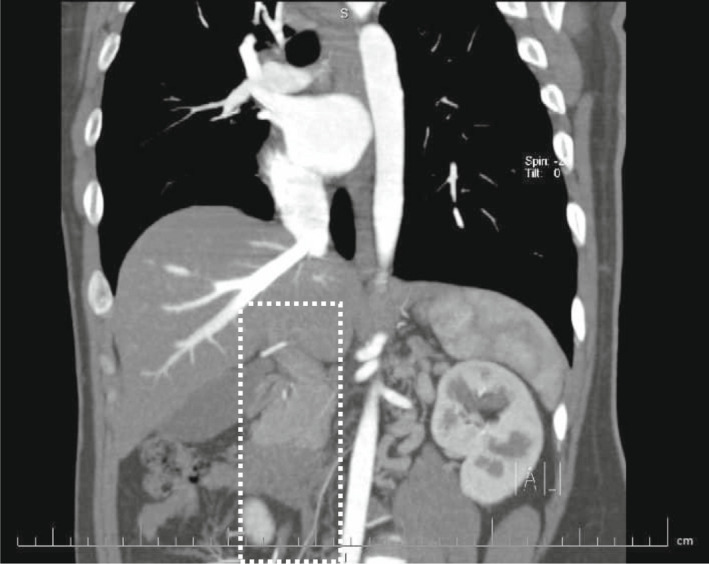
Coronal oblique reconstruction, disruption of the suprarenal and hepatic part of the IVC (dashed lines)

We decided to perform CT venography to determine the extent of venous system involvement and also its anatomy. Figure 1 shows thrombi in both common iliac veins, both external iliac veins, and right femoral vein. After convergence of common iliac veins, there is no developed IVC thereafter. In Figure 2, there are dilated collateral veins in the hilum of the kidneys with drainage into the azygos vein which is, also, dilated. Furthermore, the suprarenal IVC is not visualized in Figure 3. Findings are suggestive of azygos continuation of IVC with absent infrarenal segment. Also, there was no evidence for pulmonary embolism in the CT pulmonary angiography.

### 2.2 Treatment and follow‐up

The patient discharged on indefinite coagulation with warfarin after the international normalized ratio was within the therapeutic range between 2.0 and 3.0. In addition, compression stockings were prescribed for him. A three‐month follow‐up visit was planned to re‐evaluate the patient's clinical condition, coagulation panel and to adjust the anticoagulation dosage. After that, if his clinical status is satisfactory, we will consider longer intervals for the next re‐evaluations.

## 3 DISCUSSION

Although most patients with AIVC are asymptomatic, the most common presentation is DVT of the lower extremities. This can cause up to 5% of unprovoked DVTs in young adults.^4^, ^5^ Here, we presented a 33‐year‐old man who was referred to our tertiary center^7^ with proximal DVT in both lower extremities and the absence of the infra‐hepatic segment of IVC, in whom a trial of rivaroxaban failed.

About 15–60 different types of IVC variations and abnormalities have been reported, but only certain IVC abnormalities are of clinical significance. These variations can cause hemorrhagic events in retroperitoneal surgeries and thromboembolic events.^8^, ^9^ AIVC is one of the less frequent entities, with an incidence rate between 0.0005 and 1% in the general population.^10^ In some cases, AIVC is associated with other congenital abnormalities.^11^, ^12^ Our patient did not have any evidence of other congenital abnormalities.

There are two hypotheses about the etiology of AIVC. One suggests that the IVC did not develop during embryogenesis, either partially or completely. However, the other hypothesis discusses the possibility of early intrauterine or neonatal thrombosis of IVC that led to its regression.^13^, ^14^

To facilitate the drainage of venous blood from the lower parts of the body, collateral venous systems enlarge. The collaterals are subdivided into four main pathways: deep (azygos and hemi‐azygos), superficial (epigastric veins), portal and median (gonadal veins and pelvic plexus).^15^ In high output states, such as vigorous exercise or systemic inflammation with infections, these collateral veins may not provide enough blood flow, and some degrees of blood stasis happens. The consequent venous stasis may lead to DVT in the lower extremities. In our case, absent infrarenal segment of IVC is compensated by deep collateral pathway in the form of azygos vein continuation and by median pathways in the form of tortuous veins near the hilum of the kidneys. A similar pattern of collateral pathways is reported in another study.^16^

Thromboembolic events are one of the most frequent complications of COVID‐19 and DVT occurs in about 7.4% of non‐ICU patients.^3^ A study in Mexico showed that at presentation, up to 94% of patients with severe COVID‐19 pneumonia and even 35% of patients with mild COVID‐19 pneumonia had marked levels of inflammatory and thrombotic biomarkers such as D‐dimer (>500 ng/ml). Even after 1 month, D‐dimer remained elevated in the majority of severe cases.^17^ Furthermore, the hypercoagulable state followed by COVID‐19 may increase the probability of thromboembolic events in individuals with preexisting risk factors for VTE. Thus, it would be of benefit to screen thrombotic and inflammatory biomarkers like D‐dimer and fibrinogen in patients with risk factors for VTE to predict thrombotic risks. In our case, COVID‐19 may have triggered DVT by causing a both hypercoagulable and high‐demand state which led to venous stasis in lower venous system, but as the symptoms lasted after administration of anticoagulants and resolution of COVID‐19, we suspected other culprits.

In addition to malignancy and hereditary thrombophilia, young male patients, especially those under 30 years old, with unprovoked bilateral proximal DVTs on ultrasound should raise suspicion over the diagnosis of AIVC.^6^, ^18^ Since the Doppler ultrasound cannot visualize the IVC appropriately, in the setting of high clinical suspicion, the imaging modalities of choice are abdominal CT scan with IV contrast or magnetic resonance venography (MRV). We can determine the extent and exact variant of IVC abnormality. Also, these modalities can provide information about collateral veins and possible associated congenital abnormalities in other organs. The absence of IVC lumen alongside the presence of collateral veins on imaging studies verifies the diagnosis.

Currently, no guidelines exist to direct clinicians about the management of DVT in patients with AIVC. However, the mainstay of treatment is to avoid clot recurrence and to relieve symptoms. For the initial management of VTE, a recently published paper with a large sample size states that low‐molecular‐weight heparins (LMWH) significantly reduced mortality, recurrent VTE, and major bleedings in comparison with unfractionated heparin (UFH).^19^ In previous studies about patients with AIVC, LMWH was the most common agent for initial management.

Current guidelines of the American Society of Hematology^20^ recommends indefinite use of direct oral anticoagulants (DOAC) over vitamin K antagonists (VKA) in patients with unprovoked DVTs but states that DOACs are non‐superior to each other. Although the majority of patients were put on indefinite anticoagulation, mostly warfarin, only nine patients were taking DOACs with no adverse effects reported.^6^, ^18^, ^21^ Although multiple reports showed DVT recurrence in patients who discontinued indefinite anticoagulation or were non‐complaint,^21^, ^22^ a recent study demonstrates that the risk of VTE recurrence or leg ulcers has not changed after anticoagulation withdrawal in patients with AIVC.^23^ With these controversial data, the authors suggest a more conservative approach. Indefinite anticoagulation seems reasonable, as the underlying venous anomaly makes the patients with AIVC more susceptible to VTE recurrence.

Previous experiences with DOACs were successful in these patients.^6^, ^18^, ^21^ Esteves Cruz et al.^24^ reported that they switched from warfarin to rivaroxaban and after a 3‐year follow‐up, no thrombotic event occurred. In another study, the treatment with rivaroxaban was started but after 1 month, the symptoms persisted. Their patient was not a candidate for interventional procedures, and they opted to continue rivaroxaban indefinitely.^22^ In our case, there was another acute DVT, while the patient was taking rivaroxaban. He had full compliance with his treatment. We switched the patient to warfarin and the symptoms alleviated. A possible explanation for the failure of rivaroxaban is an underlying hepatic pathology, as elevated liver aminotransferases may be a clue for it. Also, it is possible that some pathologies affected either the structure or the function of Factor Xa, as the main target of rivaroxaban. Nevertheless, we did not investigate the patient to find hepatic pathologies and no laboratory tests was ordered to assess Factor Xa function. Further studies are warranted to investigate the use of DOACs for patients with DVT and AIVC.

Some other therapeutic options to consider is endovascular catheter‐directed thrombolysis (CDT). The current guideline recommends thrombolysis for patients with limb‐threatening DVT or younger patients with symptomatic acute DVT of iliofemoral veins before 14 days of index DVT.^20^ CDT is preferred to systemic thrombolysis, because of their lower risk for major bleedings. Several studies report successful treatment of acute DVT in patients with AIVC using CDT, with or without venoplasty and stenting.^25^, ^26^, ^27^ The surgical approach is the alternative treatment for patients with severe venous congestion, to whom both pharmacological and thrombolysis fail to respond. These methods typically involve the reconstruction of the absent segments with synthetic bypass grafts. The result is venous decompression and symptom relief.^28^, ^29^, ^30^, ^31^ Since the index DVT happened 1.5 months and it was unlikely that CDT would be of benefit for him, we decided against it.

## 4 CONCLUSION

In summary, although COVID‐19 is a major cause for thromboembolic events during the global pandemic, it should not divert our minds from other culprits. Bilateral proximal DVTs in young male patients guide the clinicians towards the diagnosis of venous anomalies like AIVC. Helical abdominal CT and MRV are used by the clinicians to confirm the diagnosis. There is no unanimous consensus about the management of AIVC‐DVT. However, indefinite anticoagulation seems to be a reasonable approach, as these patients are prone to VTE recurrence. We require more reliable documents to guide the clinicians for decision making in these patients.

## AUTHOR CONTRIBUTIONS

YJ, PG, and KH designed and formed the study conception. YJ and KH collected the clinical data of the patient. PG wrote the body of manuscript. YJ, KH, SS, and MS reviewed the draft critically. All authors read and approved the final version of the manuscript.

## CONFLICT OF INTEREST

The authors declare no conflicts of interest. We declare that we are employees of Tehran University of Medical Sciences which is a government institution, and its primary functions are medical education, medical research, medical treatment, and primary healthcare services Also, we declare that our manuscript is being submitted by the authors that “none” of them are official representative of the government.

## DATA AVAILABILTY STATEMENT

The data that support the findings of this study are available on request from the corresponding author. As the study includes medical records of an individual person, the data are not publicly available due to privacy or ethical restrictions.

## CONSENT

A written informed consent was obtained from the patient for the use of potentially identifiable images or other health information contained in our submission. Also, the patient is aware of its use and the context of such use. We understand that it is our responsibility to have secured this permission and maintain the security of such personal health information.

## References

[1] Penaloza‐Martinez E , Demelo‐Rodriguez P , Proietti M , et al. Update on extended treatment for venous thromboembolism. Ann Med. 2018;50(8):666‐674. 30345825 10.1080/07853890.2018.1538564

[2] Tagalakis V , Patenaude V , Kahn SR , Suissa S . Incidence of and mortality from venous thromboembolism in a real‐world population: the Q‐VTE study cohort. Am J Med. 2013;126(9):832 e813‐832 e821. 10.1016/j.amjmed.2013.02.024 23830539

[3] Suh YJ , Hong H , Ohana M , et al. Pulmonary embolism and deep vein thrombosis in COVID‐19: a systematic review and meta‐analysis. Radiology. 2021;298(2):E70‐E80. 33320063 10.1148/radiol.2020203557 PMC7745997

[4] Chee YL , Culligan DJ , Watson HG . Inferior vena cava malformation as a risk factor for deep venous thrombosis in the young. Br J Haematol. 2001;114(4):878‐880. 11564079 10.1046/j.1365-2141.2001.03025.x

[5] Gayer G , Luboshitz J , Hertz M , et al. Congenital anomalies of the inferior vena cava revealed on CT in patients with deep vein thrombosis. AJR Am J Roentgenol. 2003;180(3):729‐732. 12591684 10.2214/ajr.180.3.1800729

[6] Lambert M , Marboeuf P , Midulla M , et al. Inferior vena cava agenesis and deep vein thrombosis: 10 patients and review of the literature. Vasc Med. 2010;15(6):451‐459. 21183652 10.1177/1358863X10391355

[7] Poorhosseini H , Abbasi SH . The Tehran heart center. Eur Heart J. 2018;39(29):2695‐2696. 30289514 10.1093/eurheartj/ehy369

[8] Mathews R , Smith PA , Fishman EK , Marshall FF . Anomalies of the inferior vena cava and renal veins: embryologic and surgical considerations. Urology. 1999;53(5):873‐880. 10223477 10.1016/s0090-4295(99)00007-2

[9] Spentzouris G , Zandian A , Cesmebasi A , et al. The clinical anatomy of the inferior vena cava: a review of common congenital anomalies and considerations for clinicians. Clin Anat. 2014;27(8):1234‐1243. 25042045 10.1002/ca.22445

[10] Minniti S , Visentini S , Procacci C . Congenital anomalies of the venae cavae: embryological origin, imaging features and report of three new variants. Eur Radiol. 2002;12(8):2040‐2055. 12136323 10.1007/s00330-001-1241-x

[11] D'Aloia A , Faggiano P , Fiorina C , et al. Absence of inferior vena cava as a rare cause of deep venous thrombosis complicated by liver and lung embolism. Int J Cardiol. 2003;88(2–3):327‐329. 12714222 10.1016/s0167-5273(02)00404-7

[12] Rughani P , Yeung F , Halgren CR , Cada M , Schwartz S . Kidney and Inferior vena cava abnormalities with Leg Thromboses (KILT) syndrome: a case report and literature review. Paediatr Child Health. 2020;25(5):273‐275. 32765161 10.1093/pch/pxy170 PMC7395319

[13] Ramanathan T , Hughes TM , Richardson AJ . Perinatal inferior vena cava thrombosis and absence of the infrarenal inferior vena cava. J Vasc Surg. 2001;33(5):1097‐1099. 11331855 10.1067/mva.2001.114205

[14] Alicioglu B , Kaplan M , Ege T . Absence of infrarenal inferior vena cava is not a congenital abnormality. Bratisl Lek Listy. 2009;110(5):304‐306. 19507668

[15] Eyraud D , Riou B , Martin C , Vallet B . Circulation Hépato‐Splanchnique et Physiologie des Clampages Digestifs. Physiologie Humaine Appliquée. 2nd ed. Arnette; 2009:627‐656.

[16] Petik B . Inferior vena cava anomalies and variations: imaging and rare clinical findings. Insights Imaging. 2015;6(6):631‐639. 26373648 10.1007/s13244-015-0431-z PMC4656244

[17] Del Carpio‐Orantes L , García‐Méndez S , Sánchez‐Díaz S , Moguel K , Aguilar Silva A , Rosas‐Lozano A . Oral anticoagulation with rivaroxaban as thromboprophylaxis in patients recovered from COVID‐19 pneumonia in Veracruz, Mexico. J Anesth Crit Care Open Access. 2021;13:12‐15.

[18] Tufano A , López‐Jiménez L , Bikdeli B , et al. Inferior vena cava agenesis in patients with lower limb deep vein thrombosis in the RIETE registry. When and why to suspect. Int J Cardiol. 2020;305:115‐119. 31954590 10.1016/j.ijcard.2020.01.013

[19] Veeranki SP , Xiao Z , Levorsen A , Sinha M , Shah BR . Real‐world comparative effectiveness and cost comparison of Thromboprophylactic use of enoxaparin versus unfractionated heparin in 376,858 medically ill hospitalized US patients. Am J Cardiovasc Drugs. 2020;21:443‐452. 33313988 10.1007/s40256-020-00456-4 PMC8263404

[20] Ortel TL , Neumann I , Ageno W , et al. American Society of Hematology 2020 guidelines for management of venous thromboembolism: treatment of deep vein thrombosis and pulmonary embolism. Blood Adv. 2020;4(19):4693‐4738. 33007077 10.1182/bloodadvances.2020001830 PMC7556153

[21] Oblitas CM , Garcia‐Garcia A , Galeano‐Valle F , et al. Long‐term anticoagulant treatment in patients with inferior vena cava agenesis and deep vein thrombosis. Thromb Res. 2020;196:305‐307. 32977127 10.1016/j.thromres.2020.09.009

[22] Prado VE , Rey‐Mendoza JP , Wakefield CJ , Aqeel SB , Kumssa A . Infrarenal inferior vena cava agenesis and recurrent deep vein thrombosis: a case report and literature review. Oxf Med Case Reports. 2021;2021(1):omaa104. 33469470 10.1093/omcr/omaa104 PMC7802808

[23] Riera‐Mestre A , Romera A , Fernandez A , Corbella X . Long‐term follow‐up after anticoagulant treatment withdrawal in patients with deep venous thrombosis and inferior vena cava agenesis. Eur J Intern Med. 2014;25(9):e113‐e114. 25439101 10.1016/j.ejim.2014.10.008

[24] Cruz IE , Ferreira P , Silva R , Silva F , Madruga I . Inferior vena cava agenesis and deep vein thrombosis: a pharmacological alternative to vitamin K antagonists. Eur J Case Rep Intern Med. 2019;6(12):1310. 10.12890/2019_001310 PMC6936926 31893201

[25] Cronan JC , Gill AE , Gera A , Hawkins CM . Endovascular treatment of inferior vena cava agenesis using the azygos vein. J Vasc Interv Radiol. 2017;28(9):1270‐1271. 28841926 10.1016/j.jvir.2017.03.023

[26] Reslan OM , Raffetto JD , Addis M , Sundick S . Congenital absence of inferior vena cava in a young patient with iliofemoral deep venous thrombosis treated with ultrasound‐accelerated catheter‐directed thrombolysis: case report and review of the literature. Ann Vasc Surg. 2015;29(8):1657.e9‐15. 10.1016/j.avsg.2015.05.018 26184373

[27] Broholm R , Jørgensen M , Just S , Jensen LP , Bækgaard N . Acute iliofemoral venous thrombosis in patients with atresia of the inferior vena cava can be treated successfully with catheter‐directed thrombolysis. J Vasc Interv Radiol. 2011;22(6):801‐805. 21459610 10.1016/j.jvir.2011.01.449

[28] Tofigh AM , Coscas R , Koskas F , Kieffer E . Surgical management of deep venous insufficiency caused by congenital absence of the infrarenal inferior vena cava. Vasc Endovascular Surg. 2008;42(1):58‐61. 18238869 10.1177/1538574407306791

[29] Sagban TA , Grotemeyer D , Balzer KM , et al. Surgical treatment for agenesis of the vena cava: a single‐Centre experience in 15 cases. Eur J Vasc Endovasc Surg. 2010;40(2):241‐245. 20537571 10.1016/j.ejvs.2010.04.009

[30] Grieff AN , Shafritz R , Beckerman WE . Extravascular reconstruction of a congenitally absent inferior vena cava. J Vasc Surg Cases Innov Tech. 2020;6(4):681‐685. 33294753 10.1016/j.jvscit.2020.10.001 PMC7691540

[31] Ramos Aranda J , Ramirez Cerda C , Cohen Mussali S , Valdes FJ . Inferior vena cava agenesis: an unusual cause of deep vein thrombosis and pulmonary embolism in young adult patients. EJVES Short Rep. 2018;39:12‐15. 29988846 10.1016/j.ejvssr.2018.03.005 PMC6032991

